# The Impact of As-Built Surface Characteristics of Selective-Laser-Melted Ti-6Al-4V on Early Osteoblastic Response for Potential Dental Applications

**DOI:** 10.3390/jfb16070230

**Published:** 2025-06-23

**Authors:** Muhammad Hassan Razzaq, Olugbenga Ayeni, Selin Köklü, Kagan Berk, Muhammad Usama Zaheer, Tim Tjardts, Franz Faupel, Salih Veziroglu, Yogendra Kumar Mishra, Mehmet Fatih Aycan, O. Cenk Aktas, Tayebeh Ameri, Sinan Sen

**Affiliations:** 1 Department of Mechanical Engineering, Institute for Graduate School of Natural and Applied Sciences, Gazi University, Ankara 06570, Türkiye; 2 Additive Manufacturing Technologies Application and Research Center—EKTAM, Gazi University, Ankara 06560, Türkiye; 3 Chair for Composite Materials, Department of Materials Science, Faculty of Engineering, Kiel University, Kaiserstraße 2, 24143 Kiel, Germany; 4 Department of Orthodontics, University Hospital Schleswig Holstein, Arnold-Heller-Strasse 3, 24105 Kiel, Germany; 5 Chair for Multicomponent Materials, Department of Materials Science, Faculty of Engineering, Kiel University, Kaiserstraße 2, 24143 Kiel, Germany; 6 Kiel Nano, Surface and Interface Science KiNSIS, Kiel University, Christian Albrechts-Platz 4, 24118 Kiel, Germany; 7 Mads Clausen Institute, NanoSYD, University of Southern Denmark, Alsion 2, 6400 Sønderborg, Denmark; 8 Department of Mechanical Engineering, Faculty of Engineering, Gazi University, Ankara 06570, Türkiye

**Keywords:** TiAl6V4, Selective Laser Melting (SLM), surface topography, dental implant, osteoblast

## Abstract

This study investigates the potential of Selective Laser Melting (SLM) to tailor the surface characteristics of Ti6Al4V directly during fabrication, eliminating the need for post-processing treatments potentially for dental implants. By adjusting the Volumetric Energy Density (VED) through controlled variations in the laser scanning speed, we achieved customized surface textures at both the micro- and nanoscale levels. SLM samples fabricated at moderate VED levels (50–100 W·mm^3^/s) exhibited optimized dual-scale surface roughness—a macro-roughness of up to 25.5–27.6 µm and micro-roughness of as low as 58.8–64.2 nm—resulting in significantly enhanced hydrophilicity, with water contact angles (WCAs) decreasing to ~62°, compared to ~80° on a standard grade 5 machined Ti6Al4V plate. The XPS analysis revealed that the surface oxygen content remains relatively stable at low VED values, with no significant increase. The surface topography plays a significant role in influencing the WCA, particularly when the VED values are low (below 200 W·mm^3^/s) during SLM, indicating the dominant effect of surface morphology over chemistry in these conditions. Biological assays using osteoblast-like MG-63 cells demonstrated that these as-built SLM surfaces supported a 1.5-fold-higher proliferation and improved cytoskeletal organization relative to the control, confirming the enhanced early cellular responses. These results highlight the capability of SLM to engineer bioactive implant surfaces through process-controlled morphology and chemistry, presenting a promising strategy for the next generation of dental implants suitable for immediate placement and osseointegration.

## 1. Introduction

Additive technologies are a prominent trend with the potential to overhaul manufacturing practices in many fields. Dental implant manufacturing has been revolutionized as a result of such additive manufacturing technologies, especially Selective Laser Melting (SLM). SLM enables the precise fabrication of complex, patient-specific implants directly from metal powders, such as titanium and its alloys [1]. This technology allows for highly customized implants tailored to a patient’s unique anatomy, enhancing fit and promoting osseointegration, which is crucial for long-term success [2]. SLM gives us outstanding design flexibility, which enables the production of porous structures resembling natural bone, fostering tissue regeneration and decreasing the implant weight [3]. Moreover, it decreases the material waste and speeds up production, making it a financially viable solution in modern dentistry [4]. Considering the superior mechanical properties and biocompatibility, SLM is increasingly emerging as the method of choice for producing dental implants [5].

SLM has heralded a new era in dental implant manufacturing by enabling the creation of designs tailored to individual patients, featuring intricate geometries and specialized surface characteristics. This groundbreaking technology allows for the production of implants that closely match the unique anatomical shapes of a patient’s bone structure, ensuring an optimal fit and enhancing initial stability. The natural surface roughness generated during the SLM process can be leveraged to improve osseointegration, potentially eliminating the need for additional surface modifications. Implants produced using SLM techniques represent a promising pathway toward the development of more efficient, personalized, and biologically compatible solutions in the field of implant dentistry [6]. Using biocompatible materials like titanium alloys, SLM makes it possible to fabricate implants that deliver outstanding primary stability and facilitate immediate osseointegration. This suggests that dental implants manufactured through Selective Laser Melting (SLM) can be rapidly produced and placed, potentially reducing the time between surgery and implant integration. The precise anatomical alignment and favorable surface characteristics of these implants may decrease the need for extensive bone grafting or prolonged recovery periods, thereby streamlining the overall treatment process and improving patient outcomes [7]. Additionally, the porous or textured surfaces created by SLM further accelerate bone growth, enhancing the immediate stabilization of the implant [8]. The timely use of as-built SLM dental implants after surgery can accelerate the healing process, reduce the need for frequent clinical visits, and enhance overall patient satisfaction. This innovative method features implants crafted for a precise anatomical fit and optimized surface properties that facilitate early osseointegration. This represents a significant advancement in dental implant technology and personalized treatment strategies [9].

Reducing surface roughness is a critical area of research in many additive manufacturing applications due to the substantial cost associated with surface post-processing [10]. Conversely, a high degree of surface roughness is frequently desired because of its substantial influence on how biological systems respond to different materials. This underscores the essential role of the surface topography—including the morphology, dimensions, and texture—in these interactions [11]. During the early stages of the cell–material interaction, factors such as surface topography and chemistry affect cell adhesion. These attributes are closely associated with the biocompatibility of the material [12].

The surface roughness of as-built SLM dental implants plays a critical role in their performance and clinical success, influencing factors such as osseointegration, cellular response, and mechanical stability [13]. A rough or porous surface, which can be finely tuned through SLM, has been observed to positively impact early bone cell attachment, proliferation, and differentiation. These factors are crucial for achieving strong primary stability and effective osseointegration [14]. The roughness created by SLM enhances the bone–implant contact, leading to faster osseointegration and shortened healing times [15]. Additionally, SLM can create micro- and nano-structured topographies that mimic natural bone features, maximizing the surface area for bone interlocking and stimulating cellular activity at multiple levels [16]. Optimizing surface characteristics is especially important for dental implants in immediate-loading scenarios, as it helps them endure functional forces early on, minimizing micromovement and ensuring successful integration [17]. By finely tuning the surface morphology, SLM plays a crucial role in enhancing stability, accelerating recovery, and improving the long-term success of dental implants [18].

In SLM, various factors, including the choice of material, particle size, laser parameters, scanning orientation, and speed, determine the surface morphology and the roughness [19]. The laser power is the primary factor determining the extent of the surface temperature variation, significantly impacting the melting and the subsequent solidification [20]. The scanning speed is another key factor in determining the energy input for melting, which directly affects the surface quality. The laser scanning orientation and hatch spacing also play an essential role in the surface topography. Increasing the scanning speed reduces the energy input per unit area, leading to the incomplete melting of metallic particles, resulting in a rough surface finish with a higher porosity and potential defects due to the insufficient fusion between layers. Additionally, the reduced thermal energy at high speeds can cause surface texture irregularities. Conversely, decreasing the scanning speed enhances the energy density, allowing for better melting, smoother surfaces, and improved interlayer bonding, though excessively low speeds may lead to overheating, causing defects like balling or keyhole formation [21].

For instance, employing a low laser power (100–150 W) alongside high scanning speeds (800–1200 mm/s) often results in insufficient energy delivery, which can lead to the incomplete melting of particles. This incomplete melting is characterized by partially melted particles that display noticeable micro-voids and inclusions, contributing to a rougher surface texture [22,23]. A moderate laser power (200–250 W) and intermediate scanning speeds (600–800 mm/s) achieve a balanced energy input, leading to improved powder fusion and smoother surfaces with fewer defects. A high laser power (300–350 W) and slower scanning speeds (200–400 mm/s) result in much smoother surfaces due to thorough melting and longer exposure times, but excessive heat can cause thermal stresses, leading to oxidation, increased porosity, or microcracks, potentially reducing the fatigue performance and corrosion resistance [24].

In contrast, utilizing an extremely high laser power (exceeding 350 W) in conjunction with very slow scanning speeds (below 200 mm/s) can result in over-melting. This phenomenon leads to keyhole porosity, as the excessive energy vaporizes sections of the material, resulting in the formation of deep pores and inhomogeneously distributed surface features [25]. In general, such defects are not desired for various industrial applications. However, surface defects like the “stair effect” and the “balling effect” can be advantageous for creating the surface of dental implants [13]. These defects may encourage the development of additional roughness, resulting in hierarchical structures that are highly sought after for promoting a cellular response. Therefore, a different strategy should be followed in choosing the proper SLM process parameters for manufacturing dental implants compared to other SLM applications.

TiAl6V4 is the most preferred metal alloy for dental and orthopedic implants due to its mechanical strength and biocompatibility [26]. On the other hand, it is crucial that we actively manage both the surface topography and surface chemistry of TiAl6V4 to ensure optimal performance. Numerous studies have been conducted on controlling the surface topography in the SLM process. There is limited literature exploring the surface composition of TiAl6V4 parts fabricated by SLM, specifically regarding their biocompatibility and effects on cell growth, proliferation, and differentiation. When TiAl6V4 particles are melted and cooled rapidly during the SLM process, their surface chemistry may differ from parts manufactured using conventional methods such as machining, casting, or forging [27]. It has been observed that the segregation of Al can occur during the SLM of TiAl6V4 due to the rapid melting and cooling of the alloy [28]. This segregation of alloying elements has the potential to significantly alter the surface chemistry of the alloy by changing its surface oxide composition. Clinical studies have shown that the compositional variations on the TiAl6V4 surface interfere with cellular interaction and differentiation [29].

Controlling the surface chemistry of TiAl6V4 during the SLM process presents notable challenges due to the complex interactions between the Ti alloy, high-energy laser, and the environment [30]. The elevated temperatures produced by the laser can cause the TiAl6V4 particles to melt. However, rapid cooling may lead to surface oxidation, especially when residual oxygen or nitrogen is present in the build chamber. This adjustment could alter the surface composition of TiAl6V4, which could compromise its biocompatibility and resistance to corrosion [31]. Despite using inert gases like argon to regulate the atmosphere, inconsistencies in the surface chemistry can result from even minor changes in gas purity or processing conditions. Additionally, the localized thermal gradients caused by SLM could induce phase transformations or result in an inhomogeneous chemical distribution, further complicating the control of surface properties [24].

Despite the growing interest in SLM for dental implant production, the relationship between as-built surface features and an early cellular response remains insufficiently understood. This study aims to bridge that gap by investigating how variations in the laser scanning speed—and the corresponding changes in the volumetric energy density (VED)—influence the as-built surface roughness, topography, chemistry, and wettability of Ti-6Al-4V samples. By analyzing both the micro- and nanoscale surface characteristics without post-processing, we assess their role in modulating the adhesion and proliferation of MG-63 osteoblast-like cells, highlighting the functional relevance of native SLM surfaces for implant applications.

## 2. Materials and Methods

### 2.1. Sample Preparation

The TiAl6V4 (grade 5 titanium alloy) powder was sourced from General Electric (GE). This powder was produced using the proprietary Advanced Plasma Atomization (APATM) process. The mean particle diameter defined as the D50 (which represents a value at a cumulative percentage of 50% powders on a volume basis) is 44 μm. The fabrication of cuboidal TiAl6V4 components (10 mm × 10 mm × 5 mm) was conducted using an automated Selective Laser Melting (SLM) system (ERMAKSAN ENAVISION 250, Nilüfer, Bursa, Turkey). The samples were prepared with a constant laser power of 350 W and a wavelength of 1070 nm. A multidirectional laser scan strategy was used to reduce residual stress, rotating the laser scan direction by 67° for each layer. The components were constructed in a machine chamber preheated to 100–110 °C. During the build process, the chamber was maintained under an argon atmosphere to ensure an inert environment. The initial oxygen concentration ranged from 100 ppm to 2000 ppm and was reduced to 5 ppm. The depth of the TiAl6V4 powder for each sample was maintained at 60 µm, with a consistent hatch spacing of 120 µm. Four distinct types of SLM samples were produced using following VEDs: 50 W·mm^3^/s, 100 W·mm^3^/s, 200 W·mm^3^/s, and 300 W·mm^3^/s (at scanning speeds of 1.200 mm/s, 740 mm/s, 380 mm/s, and 240 mm/s, respectively). Additionally, a machined Ti6Al4V plate was used as the control substrate to represent a standard, untreated surface. This allowed us to directly compare surface characteristics and biological responses between the as-built SLM-treated surfaces and a conventional implant-grade surface.

### 2.2. Surface Characterization

Scanning electron microscope (SEM) (Zeiss Supra 55 V P, Carl Zeiss AG, Oberkochen, Germany) operating at an extra-high voltage (EHT) power supply of 5 kV was used to obtain images of the surface morphology of the SLM samples. The samples were securely mounted on aluminum stubs using carbon cement to ensure stability during imaging.

The surface roughness profiles (Ra, arithmetic average of surface roughness) were measured using a profilometer and atomic force microscopy (AFM, WITec, Ulm, Germany). While the profilometer revealed the macro-roughness at a scanning length of 1 mm, AFM analyzed the micro-roughness at a scanning area of 25 µm × 25 µm. The roughness values were measured in two directions: along the laser scan direction and perpendicular to the scan direction.

Water contact angle (WCA) measurements were conducted using a semi-automated contact angle goniometer (OCA 30, Dataphysics, Filderstadt, Germany). For static WCA analysis, 2 μL deionized (DI) water droplets were deposited on the surfaces. Advancing and receding contact angles were determined by dynamically adjusting droplet volume: DI water was incrementally added via syringe to measure advancing angles, and then withdrawn to measure receding angles, with the process videotaped for frame-by-frame analysis. During measurements, the test plate’s vertical position was carefully adjusted to maintain optimal droplet contact and imaging conditions.

The surface chemical composition of the samples was investigated using an X-ray photoelectron spectroscopy (XPS) UHV system (PREVAC Sp. z o. o., Rogów, Poland) equipped with an Al-anode (300 W). In a constant analyzer energy mode, survey scans were performed with 3 iterations, and spectra were collected at a pass energy of 200 eV. High-resolution scans were conducted at 20 iterations and the corresponding spectra were obtained at a pass energy of 50 eV. The XPS spectra were analyzed using CasaXPS software (version 2.3.23) and the Shirley algorithm was employed for background correction. Charge correction was carried out by fitting the C 1s peak with the binding energy set to 284.8 eV. Corresponding adjustments were made to all other spectra.

### 2.3. Biological Analysis

Cell cultivation and analysis were conducted according to the technique described by Naujokat et al. [32]. Osteoblast-like MG-63 cells (ATCC, Manassas, VA, USA) were grown in 100 mm dishes using a medium composed of 89% Dulbecco’s modified Eagle’s minimum essential medium (DMEM, PAA Laboratories, Pasching, Austria) supplemented with 10% fetal calf serum (FCS, Biochrom, Berlin, Germany), and 1% Penicillin/Streptomycin (Biochrom, Berlin, Germany). The cells were incubated at 37 °C with 5% CO_2_ for a period ranging from 6 to 9 days. Cell counts were conducted utilizing an inverted microscope and a hemocytometer containing 10 µL of cell suspension. Prior to biological analysis, 1 mm-thick plates were sectioned from cuboidal Ti-6Al-4V components (10 mm × 10 mm × 5 mm) initially fabricated by SLM. These sectioned SLM samples and the control substrate underwent sterilization through immersion in 70% ethanol for 10 min, followed by rinsing with phosphate-buffered saline (PBS, Sigma, St. Louis, MO, USA), before being placed into 24-well culture plates. For the seeding process, MG-63 cells that had been cultured in the larger dishes were trypsinized using a solution of 0.25% trypsin EDTA, and, subsequently, 3 × 10^4^ cells in 1 mL of fresh medium were transferred to the wells of the 24-well plates.

Staining was performed utilizing fluorescein diacetate (FDA, Sigma-Aldrich, St. Louis, MO, USA) to assess cell viability and proliferation. Following a 24 h culture period, the cells were rinsed with PBS and treated with the FDA solution. This solution comprised 30 μL of FDA stock (1 mg FDA/mL acetone (Carl Roth, Karlsruhe, Germany)) diluted in 10 mL PBS. The cells were then incubated for 20 min at 37 °C in a dark environment. After incubation, the FDA solution was removed and replaced with 500 μL of PI stock solution (1 mg PI/mL distilled water) diluted in 10 mL of PBS. Following a brief incubation of 2 min, the cells underwent two washes with PBS. Within one-hour post-staining, the cells were observed using a fluorescence microscope (Axioplan2, ZEISS, Oberkochen, Germany) and captured using a digital camera (AxioCam MRc5,ZEISS, Oberkochen, Germany). The stained cells were excited at 488 nm, while fluorescence emission from FDA was detected at 530 nm.

The proliferation of MG-63 cells in vitro was evaluated through the MTT and BrdU assays after a 24 h incubation period, adhering to the manufacturer’s instructions. Cell proliferation was quantified using the MTT Cell Proliferation Kit (Roche Diagnostics, Mannheim, Germany). For this process, 96-well microtiter plates were set up with 5 × 10^3^ cells in each well and allowed to incubate for 24 h. Following this incubation, a 100 μL eluate sample was extracted to measure cell proliferation, with optical density readings taken photometrically at a wavelength of 450 nm. Additionally, the number of proliferating cells was determined utilizing the BrdU (Bromodeoxyuridine) Cell Proliferation ELISA kit (Roche Diagnostics, Mannheim, Germany). Similarly, another set of 96-well microtiter plates contained 5 × 10^3^ cells per well and underwent incubation. A sample of 150 μL from the eluate was obtained for analysis, and the optical density for each sample was recorded using a microplate reader set to a wavelength of 450 nm.

## 3. Results and Discussion

**Figure 1:**
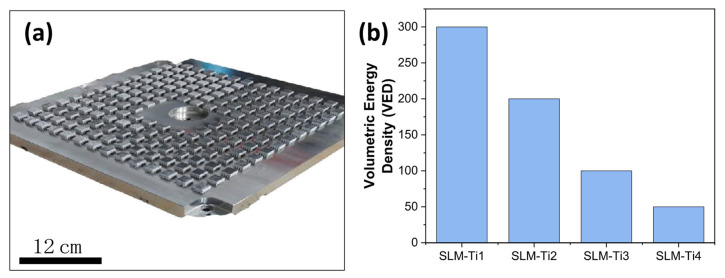
(**a**) Arrangement of SLM samples on a custom-designed holder. (**b**) Volumetric Energy Densities (VEDs) for different SLM samples.

A custom sample holder (Figure 1a) was designed to fit samples measuring 10 mm × 10 mm × 5 mm while keeping all manufacturing conditions constant, except for varying the laser scanning speeds. Four types of SLM samples (SLM-Ti1, SLM-Ti2, SLM-Ti3, and SLM-Ti4) were prepared at varying VEDs (as shown in Figure 1b) by altering the laser scanning speed between 240 mm/s and 1.200 mm/s at a constant laser power of 350 W.

**Figure 2:**
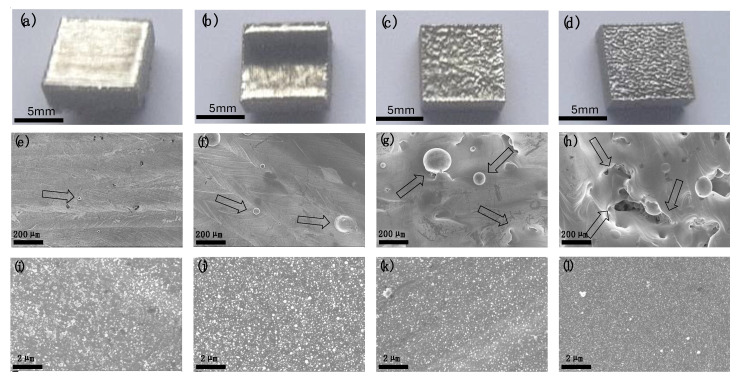
Optical images of SLM samples (SLM-Ti1, SLM-Ti2, SLM-Ti3, and SLM-Ti4) (**a**–**d**); their corresponding low magnification SEM images (**e**–**h**); and corresponding high magnification SEM images (**i**–**l**). Arrows indicates insufficient melting, lack of fusion, defects, and porosity. Visual comparisons reveal clear differences in surface morphology as a function of VEDs. SLM-Ti1 and SLM-Ti2, produced at higher energy inputs, exhibit more uniform surfaces with smoother melt tracks and fewer defects. In contrast, SLM-Ti3 and SLM-Ti4 show signs of insufficient melting and increased surface irregularities, including lack of fusion, porosity, and partially bonded particles. Opposite to the trend observed at the macro-scale, SLM-Ti4 exhibits smoother fine-scale features at the micro-scale compared to the other groups. This behavior reflects a surface-smoothing effect induced by lower VED at micro-scale during the SLM process.

Figure 2a–d show optical images of TiAl6V4 samples prepared at different scanning speeds. Figure 2e,f depicts the surface morphologies of samples as observed through SEM. The highest VED of 300 W·mm^3^/s in SLM-Ti1, resulting from the lowest scanning speed of 240 mm/sec, led to the most extensive melting of the TiAl6V4 particles. A high VED results in a narrower melt pool and more rapid melt flow, allowing for a more homogeneous material flow with a negligible porosity and enhanced fusion between the TiAl6V4 particles and layers. Consequently, the SEM image of SLM-Ti1 exhibited a smoother surface with fewer unmelted or partially melted particles and well-defined track boundaries.

Conversely, a lower VED produces a shallower and broader melt pool and sluggish melt flow, which leads to insufficient fusion between the TiAl6V4 particles and inter-layer adhesion, thus increasing the porosity. Unmelted TiAl6V4 particles can be observed in the SEM images of SLM-Ti2 prepared at a VED of 200 W·mm^3^/s (as shown by arrows in Figure 2f). SLM-Ti3, prepared at a VED of 100 W·mm^3^/s, shows an increasingly rough surface with more defects and unmelted particles (as shown by arrows in Figure 2g) due to a gradual decrease in the absorbed laser energy. As illustrated in the SEM image of SLM-Ti4, the lowest VED value of 50 W·mm^3^/s resulted in insufficient melting (partially melted particles), which caused a lack of fusion, porosity, and rougher surface (as shown by arrows in Figure 2h).

In Figure 2i–l, the surface topography of the TiAl64V4 samples is presented at a higher magnification. It is evident that smaller particles, ranging from 60 nm to 210 nm, have formed. These submicron particles cannot be categorized as unmelted or unfused TiAl6V4 particles (40–50 µm); their size clearly distinguishes them from pristine TiAl6V4 particles. The emergence of such particles seems to occur due to the reorganization of TiAl6V4 alloy particles under conditions of inhomogeneous thermal heating, a phenomenon referred to as the “balling effect”. This effect occurs when the laser-induced molten metal fails to coalesce into a uniform melt track and, instead, forms spherical droplets, or “balls”, due to surface tension forces overcoming the wetting of the substrate [33]. The balling effect in SLM can be considered a critical phenomenon for some applications, negatively impacting the quality of the fabricated parts. On the other hand, forming smaller particles through balling creates a secondary topography, which may be highly desirable for enhancing the cellular interaction on implant surfaces [34]. The shape of the balling, especially sphere and eclipse, was determined by various parameters, including the laser energy density, laser scanning speed, particle layer thickness, and the characteristics of the substrate [35].

**Figure 3:**
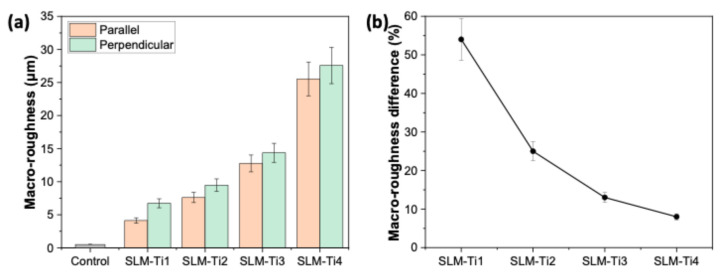
(**a**) Surface macro-roughness (Ra) values of Ti6Al4V samples fabricated by SLM at varying VEDs, compared to the polished control substrate. Results show a clear increase in surface roughness with higher VED, highlighting the influence of processing parameters on as-built surface morphology. (**b**) Percentage difference in macro-roughness measured along scanning directions parallel and perpendicular to the laser path for each SLM condition. The results emphasize the anisotropic nature of surface texture formation in SLM due to laser scanning strategy.

Long-range roughness (macro-roughness), defined by more prominent surface features that span the micrometer to millimeter scales, significantly influences cellular alignment, migration, and overall tissue organization. Cells, such as osteoblasts or fibroblasts, often orient themselves along the ridges and grooves of this roughness, promoting directional growth and tissue integration [36]. This is particularly important in applications like dental implants, where proper cell alignment and tissue formation are critical for implant success. The macro-roughness of the prepared TiAl6V4 samples was represented by the Ra values, which are depicted in Figure 3a (3D surface macro-topography analyses derived from profilometry are provided in Figure S1 to better illustrate the morphological discrepancies among SLM samples). Among the four samples, the SLM-Ti4 sample has the highest R_a_ value of 27.6 µm +/− 2.7, while the SLM-Ti1 sample exhibited the lowest roughness value of 4.1 µm +/− 0.4. These findings are in accordance with the SEM analysis shown in Figure 2h and 2e, respectively. The laser scanning speed primarily influenced the roughness values, as the laser power and hatch spacing remained constant. A lower scanning speed allowed the laser to dwell longer on a specific area, resulting in an increased energy input and the better melting and fusion of the powder. This led to smoother surfaces with the lowest roughness value, as observed in SLM-Ti1 (clearly also seen in cross-sectional SEM images given in Figure S2). Conversely, the higher scanning speed used in SLM-Ti4 reduced the laser interaction time, resulting in a lower energy input, faster cooling rates, and, consequently, increased macro-scale surface roughness (Figure S2).

As given in Figure 3b, the surface roughness shows different characteristics depending on the measurement direction with the laser scanning path (3D surface micro-topography analyses derived from AFM are presented in Figure S3 to clearly illustrate the morphological differences among the SLM samples). The surface profile displays lower roughness values when the roughness is measured parallel to the laser scanning direction. This could be due to the alignment with the melt pool tracks and the smoother contouring of solidified layers. Parallel to the laser scanning direction, the surface follows the flow of the molten material, which minimizes the detection of height variations between consecutive layers. Conversely, roughness measured perpendicular to the laser scanning direction shows higher values, possibly due to the capture of lateral discontinuities and overlaps between adjacent melt tracks. These discontinuities may arise from the inconsistent solidification during layer formation, variations in the distribution of TiAl6V4 particles, and the inherent stair-step effect linked to layer-by-layer deposition. The anisotropic roughness (high difference between the roughness measured in parallel and perpendicular to the laser scanning direction as shown in Figure 3b) is predominant for SLM-Ti1 where the VED was kept much higher than other samples. At reduced VED values, the disparity in roughness between the parallel and perpendicular orientations diminishes significantly.

**Figure 4:**
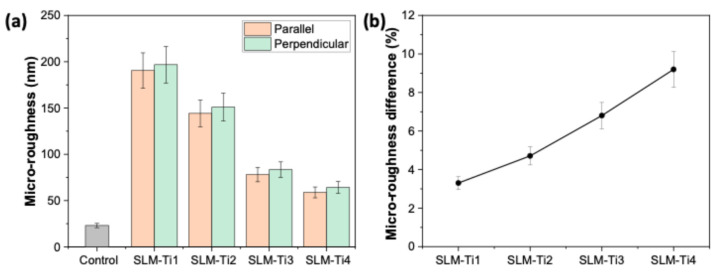
(**a**) Surface micro-roughness (Ra) values of Ti6Al4V samples fabricated via SLM at varying VEDs, compared to the control substrate. Unlike macro-roughness, micro-roughness decreases with increasing VED, indicating smoother fine-scale texture at higher energy inputs. (**b**) Percentage difference in micro-roughness measured along scanning directions parallel and perpendicular to the laser path for each SLM condition. Results demonstrate a reversed trend compared to macro-roughness, suggesting that fine-scale surface features are less anisotropic and more homogenized at higher energy levels.

It has been reported that short-range roughness (micro-roughness), typically on the micrometer to nanometer scale, affects cellular behavior at a molecular level [37]. It is crucial in enhancing cell attachment and biochemical signaling by increasing protein adsorption at the cell–material interface. We conducted an AFM analysis to reveal the micro-roughness of the prepared samples, as shown in Figure 4a. An AFM analysis demonstrated the evident variations in short-range roughness between the four types of TiAl6V4 samples, directly related to the differences in the SLM process parameters. SLM-Ti1 (with the lowest scanning speed) resulted in the roughest surface at the micro-scale, while SLM-Ti4, with the fastest scanning speed, had the lowest roughness. These findings are in accordance with high-resolution SEM images given in Figure 2l. At lower scanning speeds, the laser interacts with the material for a longer duration, resulting in an excessive heat input and more significant balling caused by the increased melt pool instability, leading to larger particle sizes. This excessive energy seems to cause the molten material to form larger droplets as it recoils and solidifies unevenly, as shown in Figure 2i. Conversely, the laser spends less time on each point at higher scanning speeds, reducing the melt pool size and heat accumulation. The inadequate melting appears to result in incomplete fusion and the formation of smaller (nanoscale) balls, as illustrated in Figure 2k,l. Such nanostructures may promote the formation of focal adhesion points, which are essential for cell proliferation, differentiation, and long-term functionality [38]. For instance, the nanoscale roughness on titanium surfaces has improved osteoblast activity, leading to better bone regeneration and faster osseointegration in dental implants [39].

**Figure 5:**
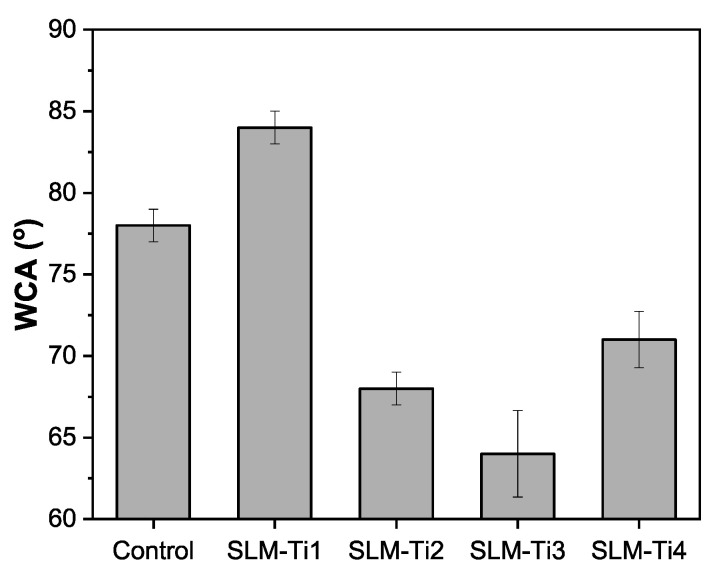
Water contact angle (WCA) measurements of Ti6Al4V samples produced via SLM at different VEDs, compared to the control substrate. The results demonstrate that as-built SLM surfaces (except SLM-Ti1) exhibit significantly reduced WCAs relative to the control, indicating enhanced hydrophilicity.

At the micro-scale, the variations in surface roughness resulting from the SLM process—whether aligned in parallel or perpendicular—are significantly reduced (see Figure 4b). In contrast, at the macro-scale, there is a pronounced difference in roughness between surfaces oriented parallel and perpendicular to the laser scanning direction (refer to Figure 3b). However, this directional dependence diminishes as the scale decreases. The rapid melting and solidification processes inherent to SLM appear to facilitate the development of nanoscale surface features that exhibit a greater uniformity across various orientations. Consequently, the nanoscale topography maintains a consistently textured appearance that is less influenced by the scanning direction. Figure 5 shows the WCA analysis and droplet profiles using deionized (DI) water, respectively. The TiAl6V4 samples prepared at different laser scanning speeds show a noticeable difference in wetting characteristics. SLM-Ti1 demonstrated the highest WCA exceeding 80°, similar to the control substrate (machined grade 5 TiAl6V4 plate). SLM-Ti1 was prepared at the lowest scanning speed, which received more energy per unit area, leading to smoother surfaces with better fusion and reduced surface porosity. When the scanning speed is reduced, we observe a significant decrease in WCA, especially for SLM-Ti2 and SLM-Ti3. Increased laser scanning speeds resulted in a higher surface roughness, reduced WCAs, and increased hydrophilicity. Oppositely, the reduced energy input increases the surface roughness, creating a microtextured surface with more peaks and valleys. These surface features seem to allow water droplets to spread more efficiently by increasing the surface area in contact with the water, reducing the WCA, and making the surface more hydrophilic. On the other hand, a high roughness exceeding a critical value can trap excessive amounts of air pockets, hindering the spreading of the water [40]. We observed a notable increase in WCA for SLM-Ti4 compared to SLM-Ti3, which we attribute primarily to the differences in surface topography. Given the nearly identical oxygen concentrations between the two samples, it is evident that morphological factors—rather than surface chemistry—seem to play a dominant role in governing wettability in this context.

While the surface topography plays a crucial role in altering the wettability of TiAl6V4, the surface chemistry is equally important. Oxidation, for instance, is an expected outcome of the SLM process, particularly in Ti alloys like TiAl6V4, leading to the formation of a passive oxide layer. This oxide layer is known to significantly enhance the hydrophilicity of the surface due to its affinity for water molecules [41]. We conducted a comprehensive XPS analysis to investigate the surface chemistry, with particular emphasis on the oxide states (refer to Figure S4). The XPS analysis of the four types of TiAl6V4 SLM samples confirmed the presence of Ti, aluminum (Al), oxygen (O), carbon (C), and a minor amount of nitrogen (N). However, detecting vanadium (V) becomes challenging when its concentration falls below 0.3%, as observed in similar studies [42]. In addition, it is known that, at high temperatures, Ti and Al oxides form preferentially due to their higher reactivity with oxygen, overshadowing the V or its oxide states [43]. Moreover, the cyclic heating and cooling during SLM processing affect the diffusion and redistribution of V, further reducing its presence in the top oxide layer [44]. Notably, the absence of V is advantageous for biomedical applications. Studies have demonstrated that V ions released from TiAl6V4 implants can hinder osteogenesis and disrupt cell differentiation [45]. Consequently, the absence of V (based on the analysis) might enhance the suitability of these samples for biomedical use.

**Figure 6:**
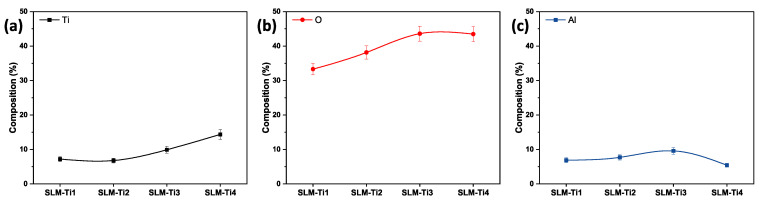
XPS-based surface elemental composition of Ti6Al4V samples fabricated via Selective Laser Melting (SLM) under varying process conditions: (**a**) titanium (Ti), (**b**) oxygen (O), and (**c**) aluminum (Al) content for samples SLM-Ti1 through SLM-Ti4. A gradual increase in surface Ti content is observed with increasing energy input. In contrast, oxygen concentration rises from SLM-Ti1 to SLM-Ti3 and plateaus at SLM-Ti4, likely due to enhanced surface oxidation during laser exposure. Aluminum levels remain relatively stable with slight variation, indicating minimal surface segregation.

XPS analyses reveal an increase in Ti content from SLM-Ti1 to SLM-Ti4, with the highest concentration observed in SLM-Ti4 at 14.36%, as shown in Figure 6a. Additionally, oxygen content rose from SLM-Ti1 to SLM-Ti3 and remained significantly elevated in SLM-Ti4 as shown in Figure 6b. This trend suggests a greater degree of oxidation on the surfaces of SLM-Ti3 and SLM-Ti4 compared to SLM-Ti1 and SLM-Ti2. The increased oxygen levels facilitate the formation of a stable oxide layer, primarily composed of TiO_2_, which markedly elevates the surface energy [46]. As a result, this modification seems to enhance the hydrophilicity, aligning with our wetting observations detailed in Figure 5.

In addition to their hydrophilic properties, the increased oxygen levels on the surfaces of Ti and its alloys are recognized to improve cell adhesion and promote biological interactions. A detailed XPS analysis revealed a characteristic doublet peak at 459.1 eV and 465 eV for all four samples, indicating that Ti predominantly exists as Ti^4+^ on the surface, as given in Figure S5. The Ti^4+^ state, present as a stable TiO_2_ layer on Ti surfaces, offers superior biocompatibility by providing a non-toxic, corrosion-resistant barrier that supports protein adsorption and cell adhesion [47]. Minor fractions of the sub-oxides Ti_2_O_3_ (457 eV and 465 eV) and TiO (454 eV and 459.1 eV) were also observed from the Ti 2p peak intensity, signifying their presence at the metal–oxide interface. A small contribution from metallic Ti (454 eV and 459.1 eV) was detected, likely due to the oxide layer being thinner than the electron penetration depth [48]. The spectral analysis of the Al in the Al 2p region indicates the possible coexistence of both metallic and oxide forms of Al in all four samples analyzed as in Figure S6. The peaks around 71 eV suggest the presence of metallic Al originating from the bulk metal alloy. Additionally, peaks near 74 eV reveal the presence of aluminum oxide (Al_2_O_3_), specifically, trivalent aluminum ions (Al^3^⁺), which are constituents of Al_2_O_3_. These Al^3^⁺ ions could be present as interstitial or substitutional ions within the TiO_2_ matrix [49]. The deconvolution of the high-resolution O 1s spectra revealed two distinct peaks, one at approximately 530.5 eV and the other at 532.3 eV, as depicted in Figure S7. The lower energy peak corresponds to Ti-O and Al-O bonds, indicating the formation of a thin surface layer composed of TiO_2_ and Al_2_O_3_. This outcome is typical for Ti alloys exposed to oxidative environments. Both types of oxides are well-known for their proven biocompatibility and non-toxic nature [50]. The peak at 532.5 eV can be attributed to several factors, including C-O bonds likely originating from surface contamination by carbon-containing species and the adsorption of water molecules [51].

**Figure 7:**
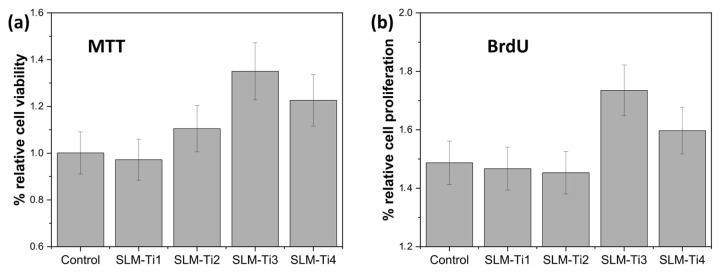
(**a**) Cell viability assessed by MTT assay for osteoblast-like MG-63 cells cultured on Ti6Al4V SLM samples and the control substrate after 24 h. Results show that all SLM-treated surfaces support high cell viability (SLM-Ti3 showed the highest viability), with no indication of cytotoxicity. (**b**) Cell proliferation assessed by BrdU assay for MG-63 cells cultured on the same samples for 24 h. Increased BrdU incorporation on select SLM-Ti3 and SLM-Ti4 surfaces indicates enhanced early-stage proliferation, highlighting the influence of as-built surface characteristics on osteoblastic response.

The cytotoxic effects of SLM samples were assessed by evaluating the response of osteoblast-like MG-63 cells after a 24 h culture period. The MTT colorimetric assay was employed for this analysis. This assay measures the ability of living cells to convert tetrazolium salt into formazan, resulting in a color change that can be quantified using spectrophotometry. Viable and healthy cells can reduce MTT, leading to a pronounced color, while damaged or dying cells exhibit minimal to no reduction, resulting in a diminished or absent color change. As illustrated in Figure 7a, the cell viability for cultures on SLM-Ti2, SLM-Ti3, and SLM-Ti4 exceeded that of the control. Notably, the viability of cells on SLM-Ti2 was comparable to that of the control, without any significant variations observed. These results indicate that the mitochondrial metabolic activity of the cell population remained unaffected after exposure to the samples, and no toxic substances were released into the supernatant, after 24 h of immersion in the culture medium. This information is vital for evaluating the safety profile of the samples for dental use.

Despite the SLM-Ti2, SLM-Ti3, and SLM-Ti4 samples demonstrating increased cell viability, SLM-Ti3 exhibited a notably superior level of cell viability. Furthermore, to gain deeper insights into the progression of the cell cycle, proliferative activity was measured through BrdU incorporation. Consistent with the MTT assay findings, SLM-Ti4 also showed the highest number of proliferating cells during a 24 h culture period. In contrast, both the control group and samples SLM-Ti1 and SLM-Ti2 displayed comparable levels of cell proliferation. This suggests that SLM-Ti3 and SLM-Ti4 offer an optimal surface topography that enhances the protein adsorption capacity of the surface while promoting cellular behaviors such as initial attachment and proliferation. The enhancement of the cell viability and proliferation on SlM-Ti3 could be attributed to the superior hydrophilicity with respect to other samples. It is important to acknowledge that variations in cell viability and proliferation may also stem from differences in surface chemistry [52].

**Figure 8:**
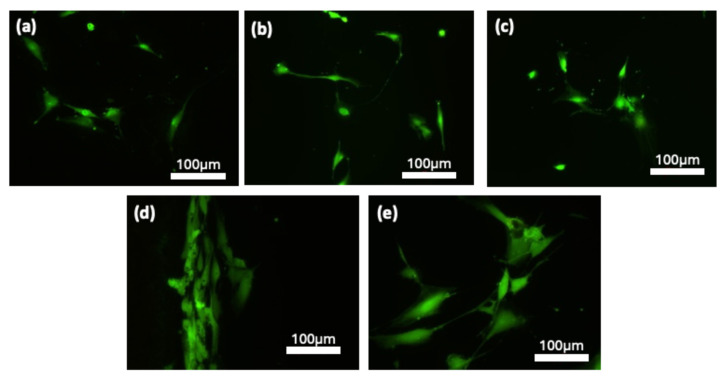
Fluorescence microscopy images of osteoblast-like MG-63 cells cultured for 24 h on Ti6Al4V samples: (**a**) machined TiAl6V4 control substrate, (**b**) SLM-Ti1, (**c**) SLM-Ti2, (**d**) SLM-Ti3, and (**e**) SLM-Ti4. Cells were stained with phalloidin to visualize F-actin, highlighting cytoskeletal organization and cell spreading. Compared to the control and lower-energy SLM surfaces, SLM-Ti3 and SLM-Ti4 exhibited the highest cell spreading and density, indicating that their dual-scale surface topography significantly enhances early cellular attachment and morphological development.

Figure 8 shows fluorescence images of adherent osteoblast-like MG-63 cells cultured on prepared SLM samples. The results showed that the MG-63 cells had a relatively well-attached and spread morphology on both SLM-Ti3 and SLM-Ti4, which is a primary indicator of cell viability. The FDA analysis revealed that there were higher cell counts on both SLM-Ti3 and SLM-Ti4 compared to those on SLM-Ti1, SLM-Ti2, and the control substrate. Notably, there was a pronounced increase in cell numbers, particularly for those cultured on SLM-Ti4 which aligns with the results of the MTT assay shown in Figure 7a. Moreover, in contrast to the other samples, the cells on SLM-Ti3 display an elongated morphology. This phenomenon is likely due to the preferential proliferation and alignment of cells, which seems to be influenced by the melt flows generated during the laser process (Figure 2c). In contrast, osteoblast-like MG-63 cells cultured on SLM-Ti1 and SLM-Ti2 exhibited distinct morphological characteristics. These cells showed limited spreading and a significant decrease in the formation of filopodia-like projections when compared to those grown on SLM-Ti3 and SLM-Ti4.

## 4. Conclusions

We demonstrated the strong potential of SLM for fabricating dental implant surfaces with inherently rough as-built topographies, potentially eliminating the need for additional post-processing treatments. By precisely adjusting the VED, SLM enables the controlled modulation of surface morphology, facilitating the generation of dual-scale roughness—macro and micro—tailored to the functional demands of dental applications. Our findings revealed that high scanning speeds (>1000 mm/s) produced surfaces with a pronounced macro-roughness (25–28 µm) and relatively low micro-roughness (50–60 nm), whereas lower scanning speeds (<250 mm/s) resulted in smoother macro features (4–4.5 µm) but significantly enhanced micro-roughness (190–220 nm). This dual-scale topography appears to promote favorable biological responses, as evidenced by the improved attachment and spreading of MG-63 osteoblast-like cells. An XPS analysis revealed that V was undetectable on the SLM-fabricated Ti-6Al-4V surfaces, likely due to its retention in the bulk, while the surface was dominated by Ti, Al, and their respective oxides. Moreover, the surface concentrations of Ti and Al deviated from the original composition of the raw Ti-6Al-4V powder, likely due to selective oxidation and surface segregation during the SLM process. Since oxygen concentrations remained relatively stable in SLM samples produced with low VED values (below 200 W·mm^3^/s), the observed variations in cellular behavior can primarily be attributed to differences in surface roughness. This dominant effect of roughness over surface chemistry was further supported by WCA measurements. Nevertheless, further investigation is required in order to elucidate the specific roles of Al and V modulating cellular responses. Future studies involving nuclear staining and high-resolution cell morphology analyses are warranted in order to better understand the mechanisms governing cell–material interactions.

## Data Availability

The original contributions presented in the study are included in the article/Supplementary Material; further inquiries can be directed to the corresponding author.

## Supplementary Materials

The following supporting information can be downloaded at: https://www.mdpi.com/article/10.3390/jfb16070230/s1, Figure S1. 3D surface macro-topography maps of SLM Ti6Al4V samples fabricated via SLM under different process conditions, (a) SLM-Ti1, (b) SLM-Ti2, (c) SLM-Ti3, and (d) SLM-Ti4, obtained through contact profilometry. Figure S2. Cross-sectional SEM images of SLM Ti6Al4V samples fabricated via SLM under varying VEDs: (a) SLM-Ti1, (b) SLM-Ti2, (c) SLM-Ti3, and (d) SLM-Ti4. Figure S3. 3D surface micro-topography maps of SLM Ti6Al4V samples, (a) SLM-Ti1, (b) SLM-Ti2, (c) SLM-Ti3, and (d) SLM-Ti4, fabricated SLM, captured using AFM. Figure S4. Wide XPS spectra of SLM samples: (a) SLM-Ti1, (b) SLM-Ti2, (c) SLM-Ti3 and (d) SLM-Ti4. Figure S5. High-resolution XPS spectra of Ti on SLM samples: (a) SLM-Ti1, (b) SLM-Ti2, (c) SLM-Ti3, and (d) SLM-Ti4. Figure S6. High-resolution XPS spectra of Al on SLM samples: (a) SLM-Ti1, (b) SLM-Ti2, (c) SLM-Ti3, and (d) SLM-Ti4. Figure S7. High-resolution XPS spectra of O on SLM samples: (a) SLM-Ti1, (b) SLM-Ti2, (c) SLM-Ti3, and (d) SLM-Ti4.
