# Supplementary material for: The Impact of As-Built Surface Characteristics of Selective-Laser-Melted Ti-6Al-4V on Early Osteoblastic Response for Potential Dental Applications
Source: J Funct Biomater. 2025 Jun 23;16(7):230. doi: 10.3390/jfb16070230 (PMC12295017; doi:10.3390/jfb16070230)
Supplement: Supplementary file 1 [file jfb-16-00230-s001.zip › jfb-3616037-supplementary.pdf]

Supplementary Materials

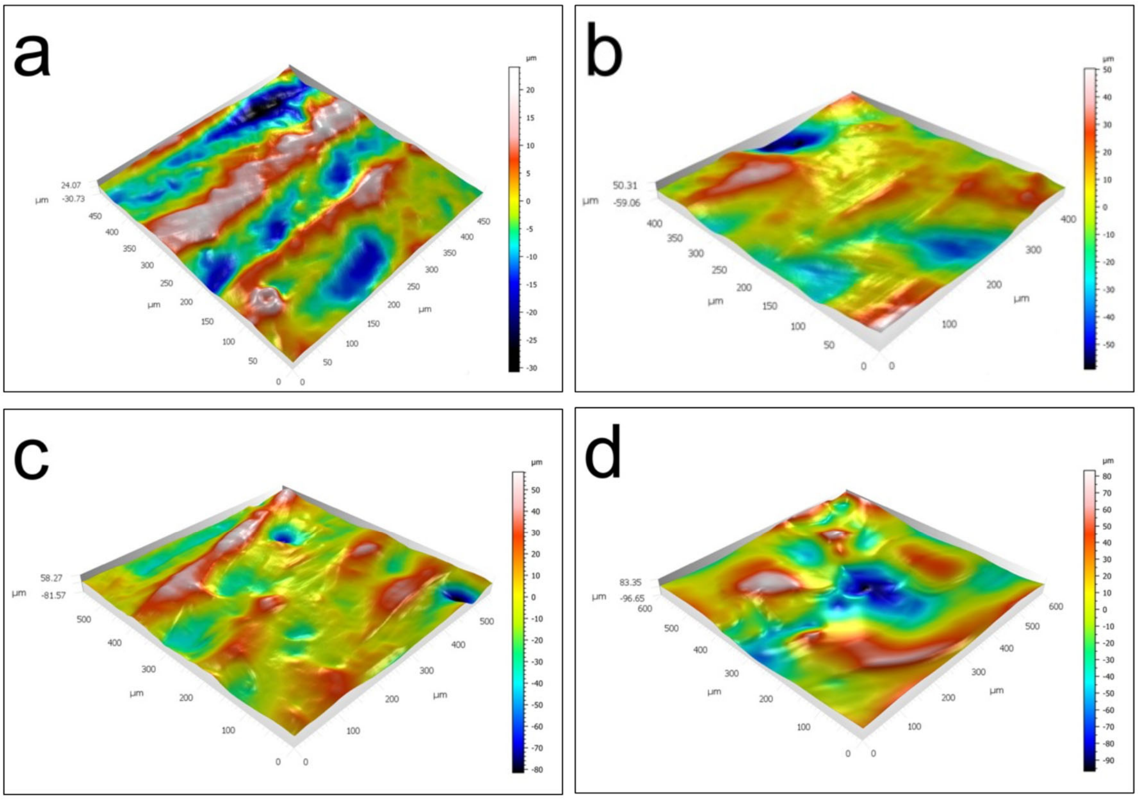

Figure S1. 3D surface macro-topography maps of SLM Ti6Al4V samples fabricated via SLM under different process conditions, (a) SLM-Ti1, (b) SLM-Ti2, (c) SLM-Ti3, and (d) SLM-Ti4, obtained through contact profilometry. These reconstructions visualize the evolution of surface morphology as a function of laser scanning speed and VED. SLM-Ti1 exhibits relatively smooth topography with shallow melt pool features, while SLM-Ti3 and SLM-Ti4 show increasingly complex and elevated surface structures, reflecting higher macro-roughness values (Ra: 26–28 μm). These differences confirm the strong correlation between energy input and surface morphology.

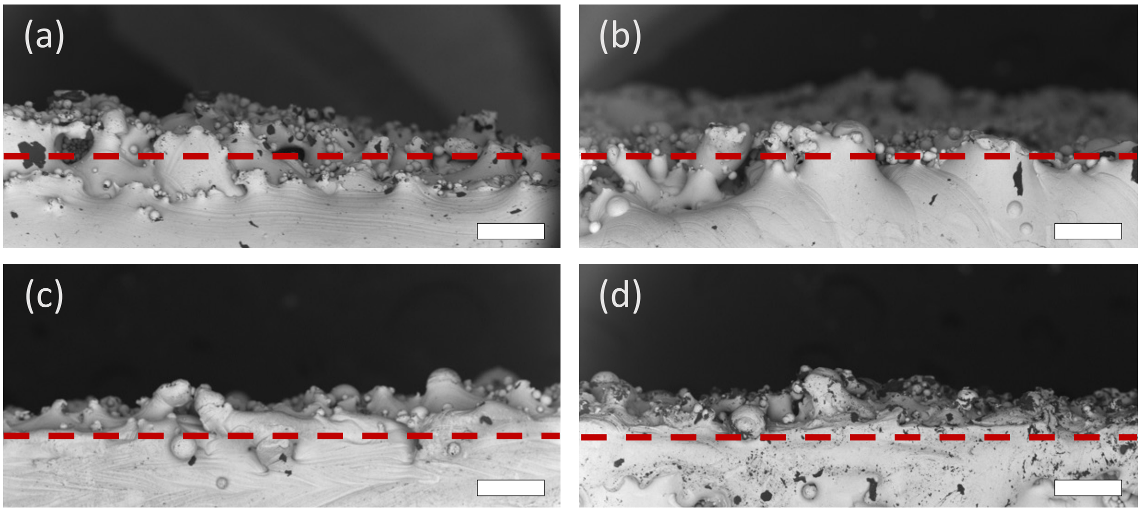

Figure S2. Cross-sectional SEM images of SLM Ti6Al4V samples fabricated via SLM under varying VEDs: (a) SLM-Ti1, (b) SLM-Ti2, (c) SLM-Ti3, and (d) SLM-Ti4 (Scale bar: 200 μm). These images illustrate the evolution of surface

morphology and melt track formation resulting from changes in VED. SLM-Ti1 and SLM-Ti2, produced with higher VEDs, show deeper melt pools and smoother cross-sectional profiles due to more extensive surface remelting. In contrast, SLM-Ti4, fabricated with the lowest energy input, displays shallower melt pools and more irregular, porous topography, indicative of incomplete fusion and lower surface energy transfer. These features correlate with the increased surface roughness observed in macro-topography analysis and suggest that lower energy input leads to more pronounced surface irregularities, which may influence cell attachment and surface wettability.

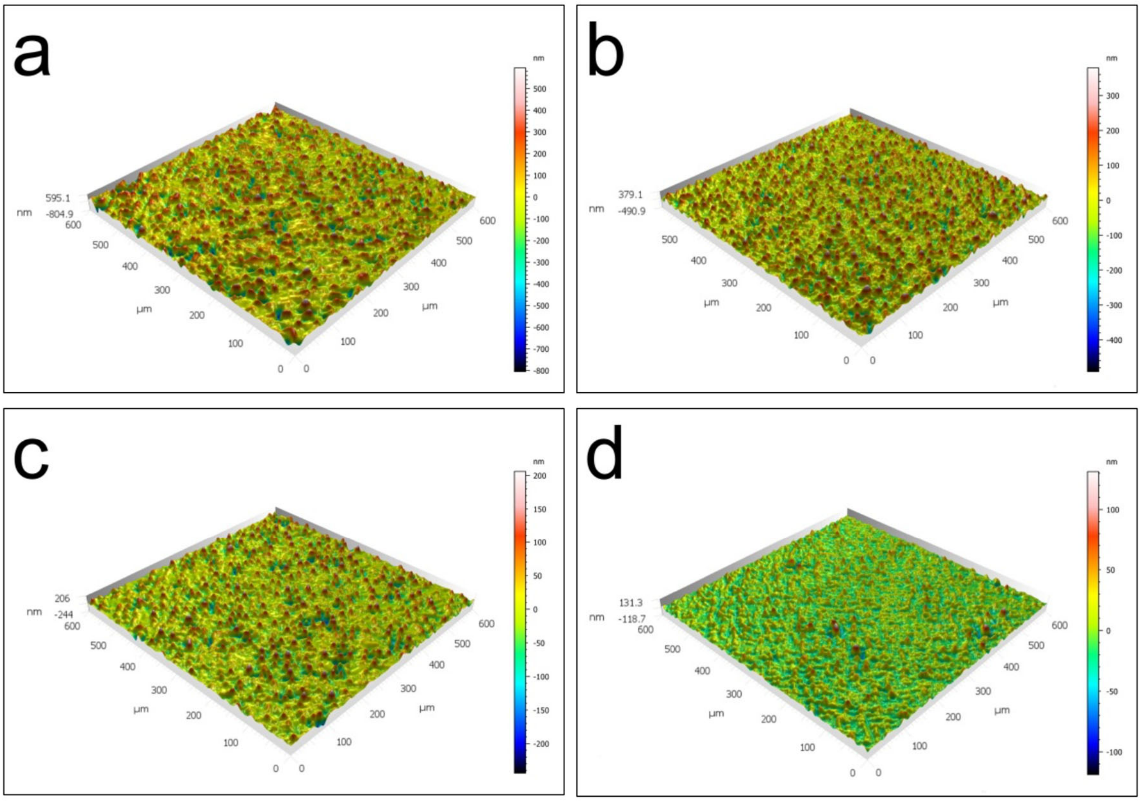

Figure S3. 3D surface micro-topography maps of SLM Ti6Al4V samples, (a) SLM-Ti1, (b) SLM-Ti2, (c) SLM-Ti3, and (d) SLM-Ti4, fabricated SLM, captured using AFM. These images highlight the fine-scale surface features at the sub-micron level and reveal a reversed trend compared to macro-topography analysis. SLM-Ti1 and SLM-Ti2 display pronounced nanoscale texture with higher micro-roughness (up to ~190 nm Ra), whereas SLM-Ti3 and SLM-Ti4 exhibit smoother micro-surfaces (Ra: 58–64 nm) due to increased laser energy input and surface melting. These results indicate that, while higher VED enhances macro-scale roughness, it concurrently reduces micro-roughness, suggesting an energy-driven smoothing effect at the nanoscale. This dual-scale modulation is critical for controlling both wettability and biological responses on as-built implant surfaces.

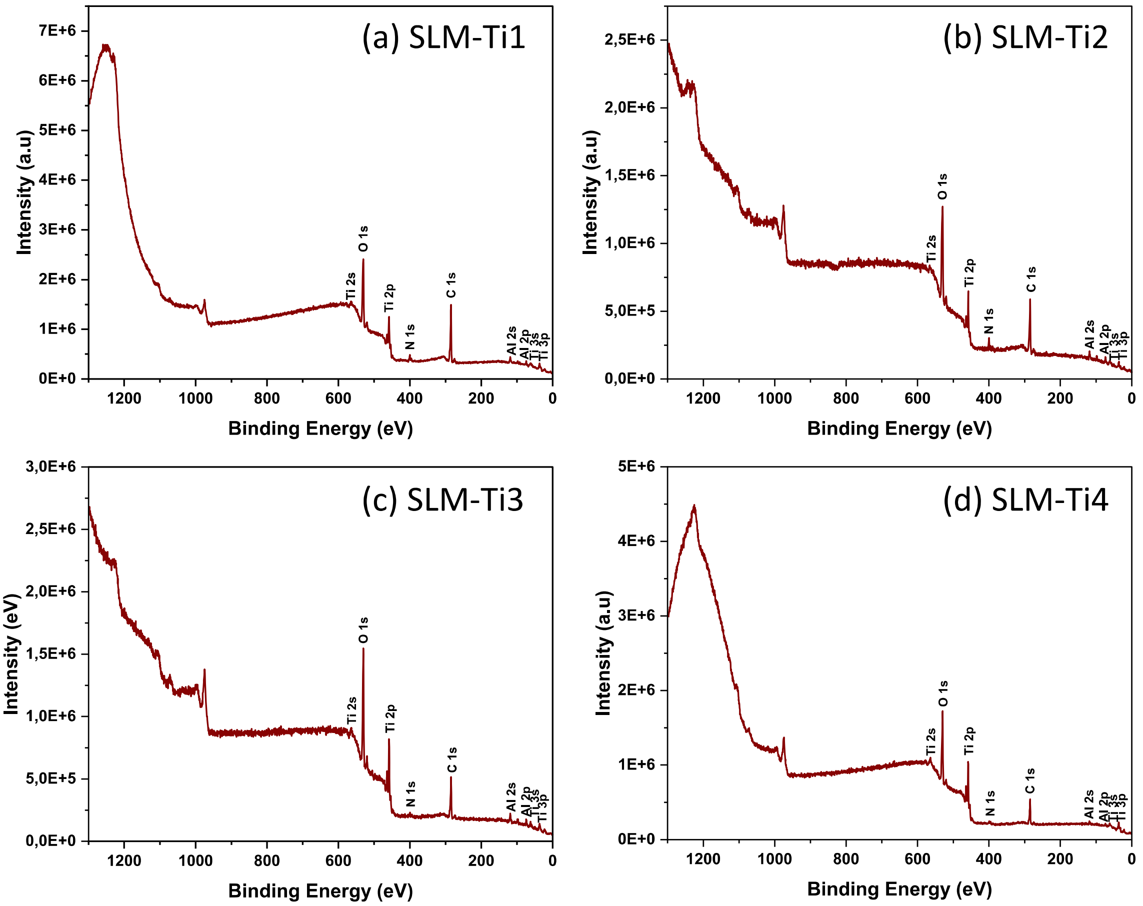

Figure S4. Wide XPS spectra of SLM samples: (a) SLM-Ti1, (b) SLM-Ti2, (c) SLM-Ti3, and (d) SLM-Ti4.

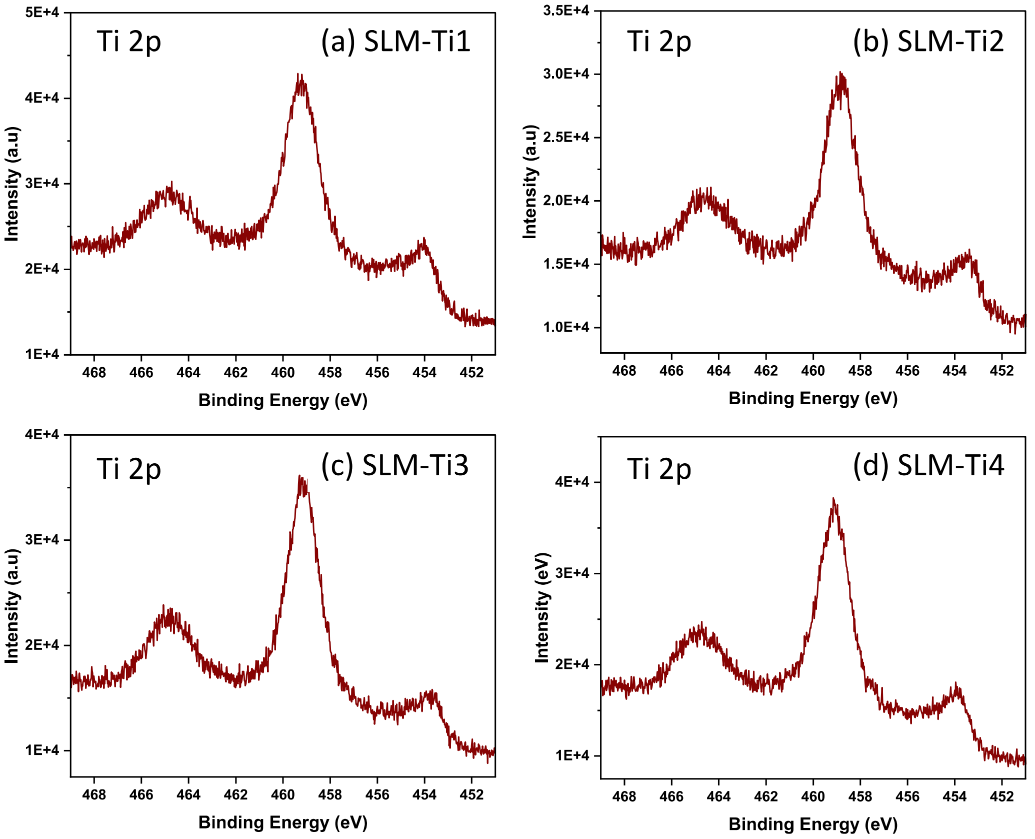

Figure S5. High-resolution XPS spectra of Ti on SLM samples: (a) SLM-Ti1, (b) SLM-Ti2, (c) SLM-Ti3, and (d) SLM-Ti4.

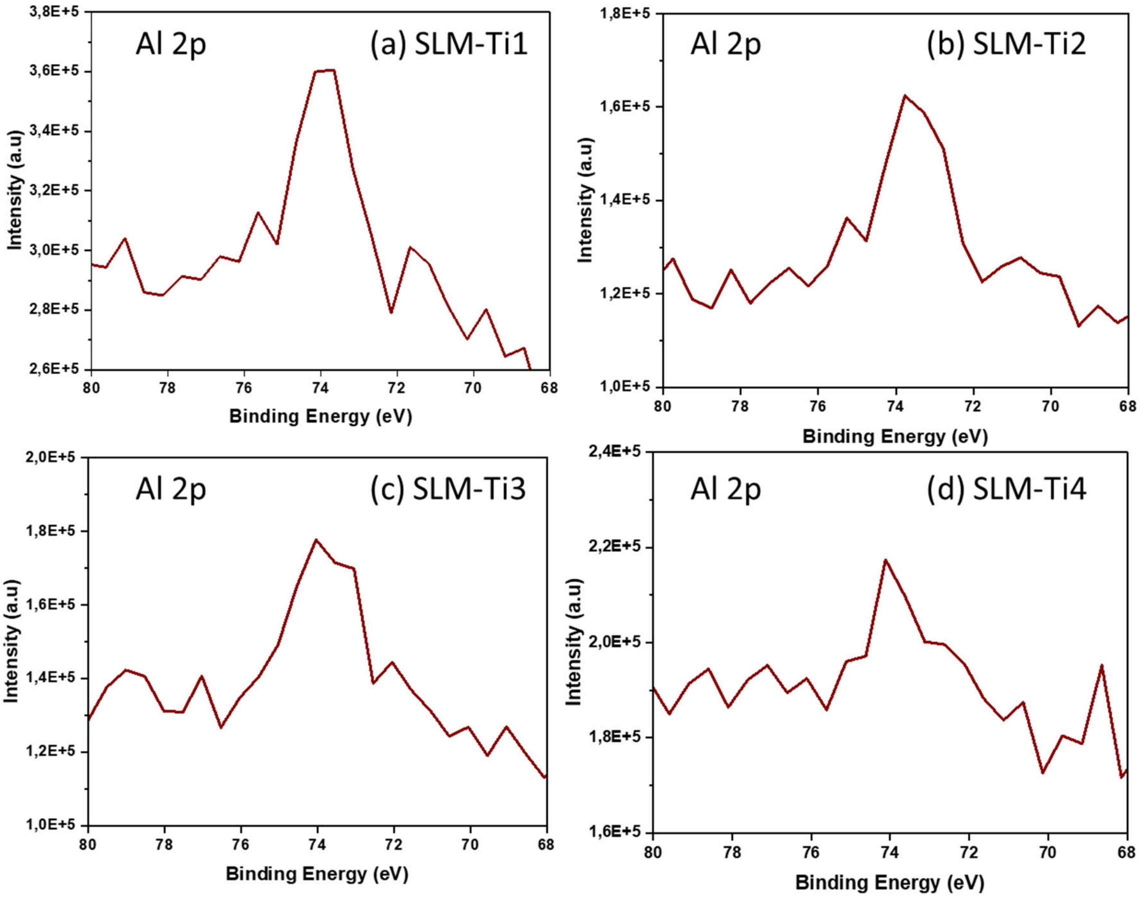

Figure S6. High-resolution XPS spectra of Al on SLM samples: (a) SLM-Ti1, (b) SLM-Ti2, (c) SLM-Ti3, and (d) SLM-Ti4.

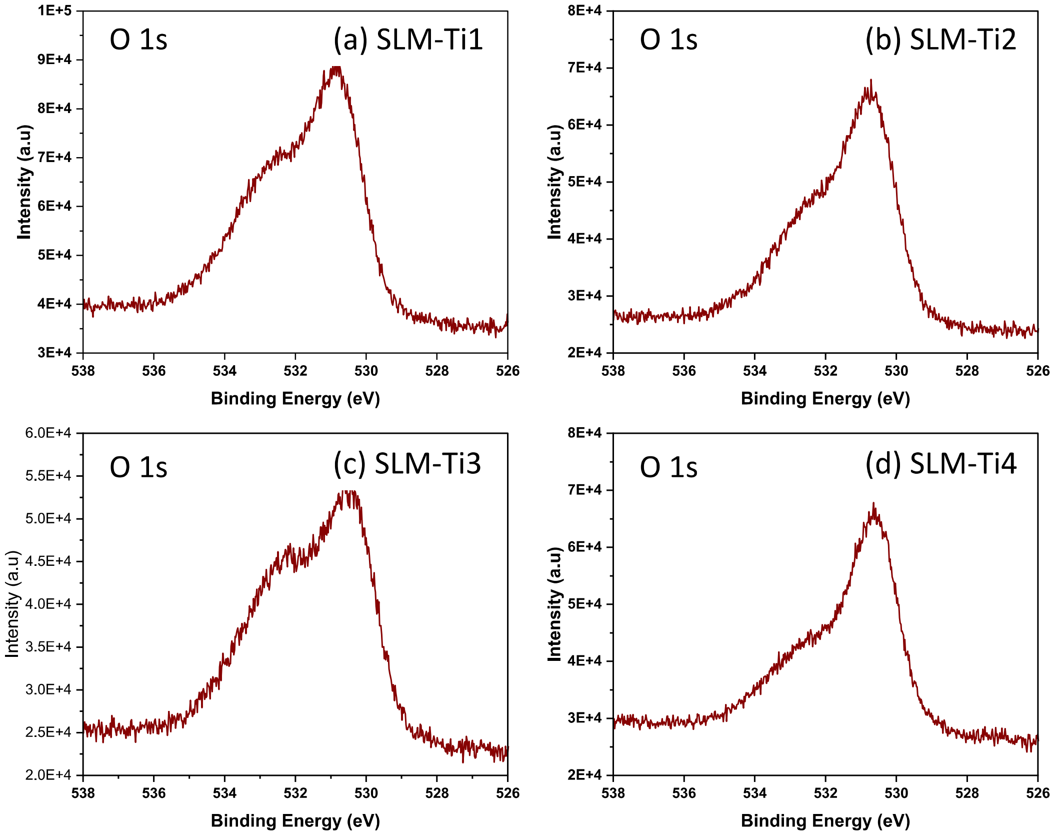

Figure S7. High-resolution XPS spectra of O on SLM samples: (a) SLM-Ti1, (b) SLM-Ti2, (c) SLM-Ti3, and (d) SLM-Ti4.
